# Cerebral aneurysm presenting with aseptic meningitis: a case report

**DOI:** 10.1186/1752-1947-7-244

**Published:** 2013-10-18

**Authors:** Muhammad Azfar Saleem, R Loch Macdonald

**Affiliations:** 1 Division of Neurosurgery, St. Michael’s Hospital, Labatt Family Centre of Excellence in Brain Injury and Trauma Research, Keenan Research Centre of the Li Ka Shing Knowledge Institute of St. Michael’s Hospital, 30 Bond Street, Toronto, ON, M5B 1W8, Canada; 2 Department of Surgery, University of Toronto, 30 Bond Street, Toronto, ON, M5B 1W8, Canada

**Keywords:** Cerebral aneurysm, Cerebrospinal fluid inflammation, Subarachnoid hemorrhage

## Abstract

**Introduction:**

This case highlights the potential importance of new-onset headache, even in the absence of other worrisome features, in a patient with a cerebral aneurysm.

**Case presentation:**

A 61-year-old Caucasian woman presented with nonspecific insidious onset of headache, a superior cerebellar artery aneurysm and cerebrospinal fluid lymphocytosis. She had a subarachnoid hemorrhage 21 days later, at which time the aneurysm had enlarged. The aneurysm was repaired endovascularly and the patient recovered with a modified Rankin score of 1.

**Conclusions:**

This case suggests that new onset of chronic headache in a patient with an unruptured aneurysm may be due to aneurysm growth and can be associated with cerebrospinal fluid lymphocytosis. Headaches are common and may occur incidentally in patients with cerebral aneurysms, but new-onset headache, even if mild, should prompt consideration for timely aneurysm repair.

## Introduction

Improvement and advancements in diagnostic tools, and their application, has increased the incidence of diagnosis of unruptured aneurysms [1]. Unruptured aneurysms have been associated with headache, cranial nerve palsies and seizures [1,2]. The most common chronic symptom is nonspecific headache. The etiology of these headaches is controversial and, in some cases, chronic or slow-onset headache may not be due to an unruptured aneurysm. We describe a patient with an unruptured superior cerebellar artery aneurysm who presented with new onset of chronic headache, normal cranial computed tomography (CT) and cerebrospinal fluid (CSF) containing lymphocytes. The aneurysm ruptured 21 days later. This case is important because it suggests that new-onset headache in a patient with an unruptured intracranial aneurysm could indicate that there is a risk that the aneurysm could rupture soon, particularly if there is evidence of inflammation in the CSF.

## Case presentation

**Figure 1:**
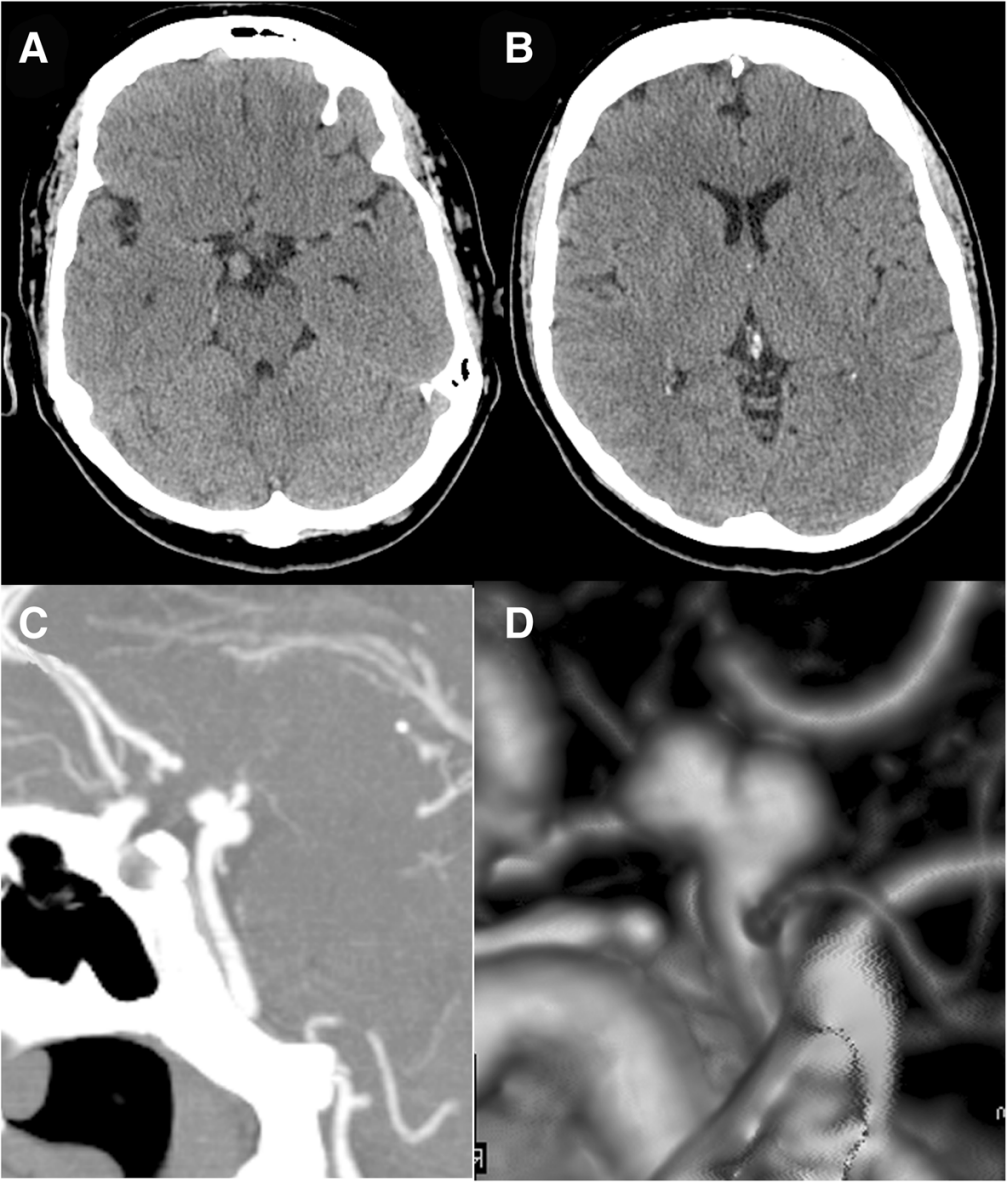
**Computed tomography at first presentation.** Cranial computed tomographic scan showing density in the right interpeduncular cistern **(A)** and no subarachnoid hemorrhage or hydrocephalus **(B)**. A computed tomographic angiogram sagittal view **(C)** and lateral view of a reconstruction **(D)** show an aneurysm arising from the basilar artery distal to the origin of the right superior cerebellar artery.

A 61-year-old, right-handed Caucasian woman presented with a two-week history of increasing headache, which was not described as severe. There was no specific time when the headache began. Her blood pressure was 130/80mmHg. A plain CT scan of the head with 5mm thick axial slices obtained on a 64-slice scanner did not show any subarachnoid hemorrhage (SAH). Her CT angiogram showed an aneurysm with a transverse diameter of 0.6cm and a maximum diameter of 0.8cm arising from the basilar artery at the origin of the right superior cerebellar artery (Figure 1). Cerebrospinal fluid obtained by lumbar puncture showed 513×10^6^/L erythroid and 293×10^6^/L nonerythroid cells (98% lymphocytes) in the first tube and 16×10^6^/L erythroid and 371×10^6^/L nonerythroid cells (92% lymphocytes) in the third tube. Her total protein level was 0.57g/L (normal range 0.15 to 0.45g/L) and glucose was 2.4mmol/L (serum was 9mmol/L). There was no xanthochromia on visual inspection of the CSF. Culture of the CSF did not yield any bacteria. Enterovirus, herpes simplex virus, Epstein-Barr virus, cytomegalovirus, human herpes virus 8 and West Nile virus immunoglobulin M ribonucleic acids were not detected by polymerase chain reaction in the CSF. A diagnosis of viral meningitis with associated right superior cerebellar artery aneurysm was made. The patient was discharged home and seen as an outpatient 20 days later, at which time she complained of fatigue and blurry vision in the right eye. There was no diplopia or pupil abnormality on examination. It was recommended that she undergo endovascular coil repair of the aneurysm, which she went home to consider.

**Figure 2:**
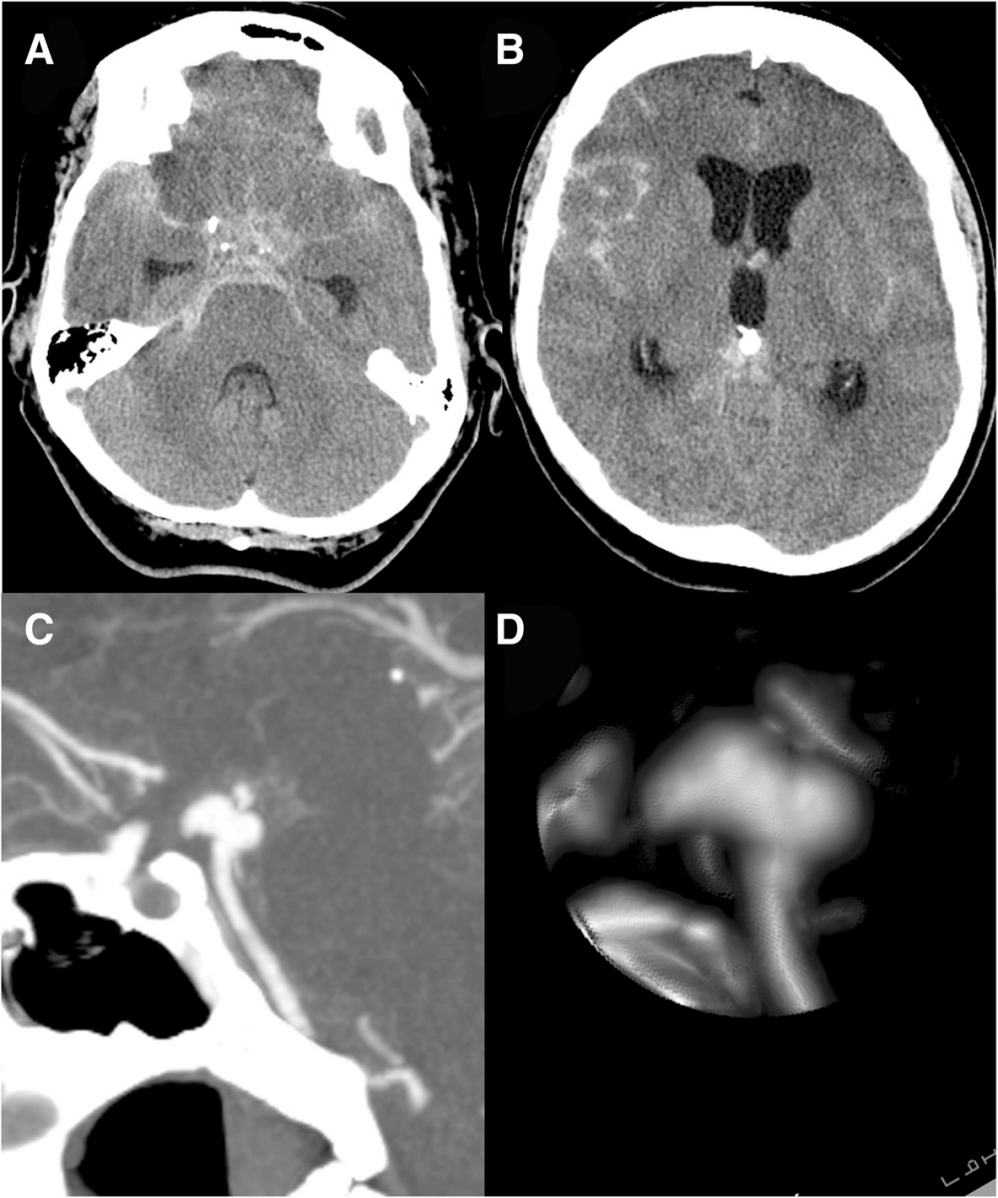
**Cranial computed tomography scan 21 days after presentation.** A cranial computed tomography scan 21 days after presentation showing diffuse subarachnoid hemorrhage **(A)** with hydrocephalus **(B)**. The computed tomographic angiogram sagittal view **(C)** and lateral view of a reconstruction **(D)** show growth of the aneurysm on the anterior aspect.

Twenty-one days after the first presentation, the patient presented with sudden onset of severe headache. Her Glasgow Coma Score was 15 and she was neurologically intact. A cranial CT scan showed diffuse, thin SAH, blood in the occipital horns of the lateral ventricles and acute hydrocephalus. Our patient deteriorated and when her Glasgow Coma Score was 10, an external ventricular drain was inserted. CT angiography showed a bilobed (anterior and posterior lobes) right superior cerebellar artery aneurysm that had slightly increased in size to approximately 0.6 by 1.0cm (Figure 2).

**Figure 3:**
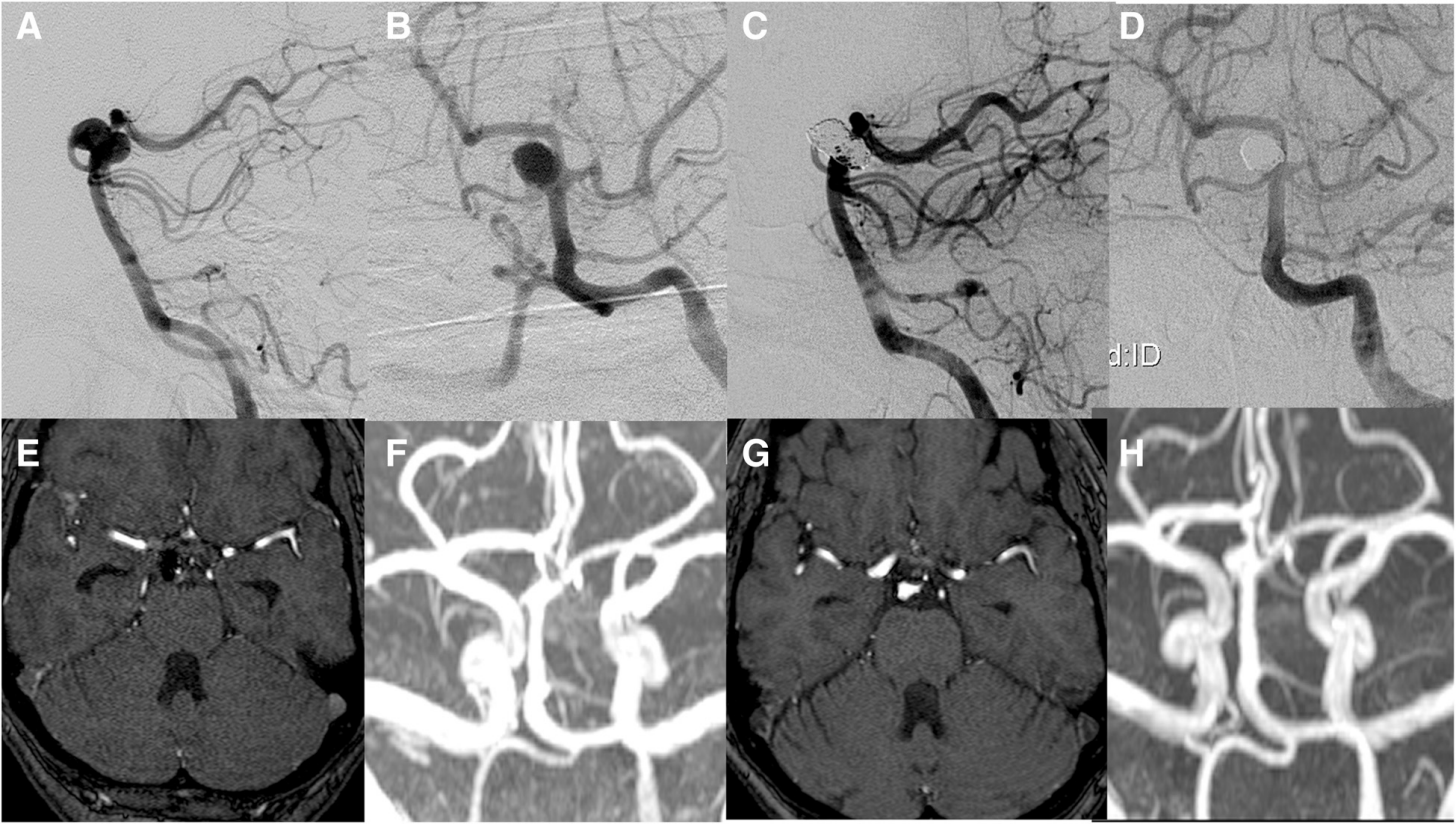
**Cerebral angiogram.** Lateral **(A)** and anteroposterior **(B)** catheter angiograms, the left vertebral artery injection showing the right superior cerebellar artery aneurysm. The aneurysm was repaired by endovascular coiling, leaving minimal or no residual neck visible on the lateral **(C)** and anteroposterior **(D)** views after treatment. Gadolinium-enhanced magnetic resonance angiography seven days later shows a small residual neck **(E, F)**. At the last follow-up 51 months later, there has been some growth of the residual neck on magnetic resonance angiography **(G, H)**.

Eighteen hours later, our patient underwent endovascular coiling of her aneurysm. This was done using balloon remodeling. There was a small residual portion of aneurysm neck filling (residual neck). Our patient recovered and at last follow-up 51 months later, had a modified Rankin score of 1. She complained of fatigue and occasional headaches but was fully functional. A magnetic resonance angiogram (MRA) showed a slight increase in the size of the residual aneurysm neck (Figure 3).

## Discussion

The question in this case is what caused our patient’s initial headache. Intracranial aneurysms can present with SAH, with other acute symptoms such as cranial nerve palsies or sudden-onset headache in the absence of SAH, seizures and focal neurological deficits, or with chronic symptoms, such as headache or cranial nerve or other focal neurological deficits [2]. This patient had two weeks of headache that was not sudden in onset or severe. It was not associated with SAH on a CT scan, although there were some erythrocytes and lymphocytes in the CSF. The differential diagnosis included SAH, viral meningitis or subarachnoid inflammation from the aneurysm. Chronic headache also is a common symptom from an unruptured aneurysm, but the etiology of these headaches is variable and sometimes unclear. This case suggests some potential causes.

One potential cause of our patient’s initial headache was SAH. We considered this less likely because there was no sudden onset of headache, no SAH seen on high-quality CT done soon after presentation and no xanthochromia. This opinion is consistent with a report of a 54-year-old man with a three-day history of progressive headache that became the worst headache of his life [3]. A CT scan showed no SAH and his CSF showed 3467×10^6^/L erythrocytes but no xanthochromia. The authors did not interpret the findings as a SAH. The patient had a right superior cerebellar artery aneurysm on catheter angiography. A magnetic resonance imaging (MRI) scan showed signal change in the aneurysm wall that was interpreted as subintimal hemorrhage. Intraoperative examination of the aneurysm during clipping also showed no SAH but did show intramural hemorrhage in the dome of the aneurysm. The present case did not have an MRI scan so we cannot determine if there was intramural hemorrhage. It is possible that the new headache and erythrocytes and lymphocytes in the CSF were due to hemorrhage into the wall of the aneurysm or minor leakage of blood into the subarachnoid space. The growth of the aneurysm over 21 days indicates that the aneurysm was actively changing. New onset of headache associated with unruptured aneurysms has focused mostly on acute presentation and has been postulated to be due to events like this such as intramural hemorrhage or thrombosis or aneurysm growth. Hemorrhage into the wall of aneurysms is reported, although more commonly with large saccular aneurysms or with dissecting or antherosclerotic fusiform aneurysms [4,5]. Importantly, headaches preceding aneurysmal SAH, as seen in this patient, have been attributed to minor hemorrhage, aneurysm growth or thrombosis or ischemia from emboli from the aneurysm [6]. A warning leak or preferably, a sentinel headache, usually defined as a sudden severe headache that precedes definitive diagnosis of SAH by days or weeks, occurs in 10% to 43% of patients [7].

Factors that suggest this could have been SAH are that up to 10% of patients with SAH do not give a history of sudden-onset headache [8]. On the other hand, there is emerging literature to suggest that high-quality CT can exclude SAH. Perry *et al*. reported that CT on third-generation or later scanners with at least four slices/rotation had a sensitivity of 93%, specificity of 100%, negative predictive value of 99% and positive predictive value of 100% for SAH in patients with normal consciousness who were investigated in emergency departments for acute headache [9]. All of these values were 100% when CT was obtained within 6 hours of the onset of headache. Another series of 137 patients who underwent CT scanning and lumbar puncture for investigation of headache and who had a Glasgow Coma Scale score of 15 found CT had a sensitivity of 99% within 6 hours and of 90% >6 hours after the ictus [10]. The conditions upon which these studies are based must be remembered, which include the time of investigation. Also in the first report, the studies were interpreted by a neuroradiologist. The studies suggest that SAH is rare or nonexistent if it is not seen on a CT scan done within 6 hours of onset of headache. The patient described here, however, did not have a defined time of headache onset.

In the case described here, there was no xanthochromia, which also makes SAH unlikely. On the other hand, xanthochromia takes time to develop after SAH and since our patient had no defined time of onset of headache, it is possible the CSF was sampled too soon after the onset of headache to detect xanthochromia. Another interpretation is that our patient did have SAH. She had erythrocytes in the CSF and any erythrocytes in the CSF are abnormal. Distinguishing a traumatic lumbar puncture from SAH, however, is sometimes problematic and there are no accepted numbers of erythrocytes in the CSF that distinguish the two. Subarachnoid hemorrhage has been considered to be present when there are more than 400×10^6^/L (400 per mm^3^) [11]. Another study considered SAH when there were more than 5×10^6^/L erythrocytes [9]. A case report concluding that an acute headache from an aneurysm was due to hemorrhage into the aneurysm wall found 3467×10^6^/L erythrocytes in the CSF [3].

Guidelines for investigation of patients presenting to an emergency department with headache and normal level of consciousness have included different combinations of criteria, including age >40 or 45, complaint of neck pain or stiffness, loss of consciousness, onset with exertion, arrival by ambulance, vomiting, diastolic blood pressure >100mmHg and systolic blood pressure >160mmHg [12]. Our patient would meet only the age criterion [12].

Another diagnosis is that our patient had viral meningitis. Some viruses are undetectable by polymerase chain reaction in the CSF. It is possible, however, that our patient had viral meningitis and that this contributed to the growth and rupture of the aneurysm.

One could hypothesize that inflammation, perhaps due to viral meningitis in the subarachnoid space induced by growth of the aneurysm, could cause this clinical scenario. We found one case report to suggest that this can occur. Kraus *et al*., reported a 50-year-old woman who developed a third nerve palsy. An MRI scan of the brain was normal and did not show an aneurysm, although it was not said whether MRA was done at this time. Lumbar puncture showed no xanthochromia, no red blood cells but nine white blood cells (97% lymphocytes) and a protein of 41g/L [13]. Three weeks later, there was enhancement of the cisternal segment of the third nerve and a posterior communicating artery aneurysm was identified on MRA. This seems to be a case where aneurysm growth in the absence of hemorrhage was associated with subarachnoid inflammation. This is a rare situation since most reports of growing aneurysms do not mention headache as a symptom and most patients are being reimaged as part of scheduled follow-up [14]. Even when they develop symptoms, headache is not universal [8]. This contrasts with studies demonstrating inflammation in the walls of unruptured and ruptured aneurysms [15].

## Conclusions

This case suggests that new onset of chronic headache in a patient with an unruptured aneurysm may be due to aneurysm growth and should prompt consideration for timely aneurysm repair.

## Patient’s perspective

Fortunately, I knew I had an aneurysm before it burst. While at our college, I had a severe headache and fever. The local Minden hospital sent me to Lindsay for a CT scan, suspecting meningitis or an aneurysm. The CT scan showed a suspicious spot on my brain and I was sent to St. Michael’s Hospital via ambulance and, after a spinal tap and another scan, it was determined I had both. After recovering from meningitis, I saw Dr. Macdonald and he suggested a committee would determine how to deal with the aneurysm in a few weeks’ time. Unfortunately, it burst a few days later. I was home, alone, and knew without a doubt that something very serious had happened because of the pain, but was able to call 911. After surgery, it took four months to recover my strength, appetite and normal bowel functions. Now, almost six years later, I still have headaches and tire easily but, for the most part, I am ‘back to normal’. As one of my doctors said, it was a ‘near-death experience’ for me and one that I think about almost daily.

## Consent

Written informed consent was obtained from the patient for publication of this manuscript and any accompanying images. A copy of the written consent is available for review by the Editor-in-Chief of this journal.

## Abbreviations

CSF: cerebrospinal fluid; CT: computed tomography; MRA: magnetic resonance angiography; MRI: magnetic resonance imaging; SAH: subarachnoid hemorrhage.

## Competing interests

RLM receives grant support from the Physicians’ Services Incorporated Foundation, Brain Aneurysm Foundation, Canadian Stroke Network and the Heart and Stroke Foundation of Ontario. RLM is a consultant for Actelion Pharmaceuticals and Chief Scientific Officer of Edge Therapeutics, Inc.

## Authors’ contributions

MAS reviewed the chart and prepared the first draft of the manuscript. RLM revised the manuscript, added the discussion and references sections and prepared the figures. Both authors read and approved the final manuscript.
